# Salinity and stable isotope dataset for Guayas estuary waters

**DOI:** 10.1016/j.dib.2021.106776

**Published:** 2021-01-19

**Authors:** Niall C. Slowey, Edwin B. Pinto

**Affiliations:** a Department of Oceanography, Texas A&M University, College Station, TX 77843 USA; b Instituto Oceanográfico de la Armada (INOCAR), Av. 25 de Julio, Guayaquil, Ecuador

**Keywords:** Guayas estuary, Oxygen isotope and hydrogen isotope ratios, Salinity, Water properties

## Abstract

The Guayas estuary is an ecologically and economically vital, large estuarine system located on the western coast of South America. A suite of ∼400 water samples was collected and analyzed to enable investigation of the sources of water types within this estuary, as well as environmental processes active within various portions of it. These samples were obtained at sites distributed across the major areas of the inner and outer portions of the estuary at the ends of consecutive dry (December/January) and rainy (May) seasons. At each site, Van Dorn bottles were lowered into the water from a boat (or bridge) and then triggered when they reached specified depths. When the Van Dorn bottles were brought back aboard the boat, aliquots of water from them were promptly sealed in glass containers for later analysis. These were transported to onshore laboratories where their salinities, and their oxygen (δ^18^O) and hydrogen (δ^2^H) isotopic compositions were measured. Established analytical procedures and standards were employed to obtain a robust set of resultant data. The samples, analyses, and data presented here support the associated research article, “Stable isotope evidence for the origins of waters in the Guayas estuary and Gulf of Guayaquil” [1], to which readers are referred for interpretation.

## Specifications Table

**Table:** 

Subject	Earth and Planetary Sciences
Specific subject area	Oceanography
Type of data	Table
How data were acquired	Water samples were collected in the field. Salinities were determined using a HACH HQ14d conductivity meter; oxygen and hydrogen isotope ratios were determined using a Picarro L2120i cavity ring-down spectroscopy (CRDS) analyzer.
Data format	Raw and analyzed data
Parameters for data collection	Water sample collection date, latitude (°), longitude (°), depth (m), salinity (PSU), oxygen isotope ratio (‰), and hydrogen isotope ratio (‰)
Description of data collection	Sampling devices were deployed from boats to collect waters at sites and water depths distributed across the Guayas estuary. Their salinities were determined from measured conductivity (±0.1 precision on PSS-78 practical salinity scale) at INOCAR, and their stable isotope ratios of oxygen (δ^18^O) and hydrogen (δ^2^H) were measured at Texas A&M University using a CRDS analyzer at (precisions of individual δ^18^O and δ^2^H analyses relative to VSMOW are ±0.1‰ and ±1.0‰, respectively).
Data source location	Water samples were collected in the Guayas estuary of Ecuador (see Table 1 for specific coordinates). The samples were analyzed at these institutions:
	1) Marine Chemistry Laboratory
	Instituto Oceanográfico de la Armada (INOCAR)
	Av. 25 de Julio
	Guayaquil
	Ecuador
	2) Stable Isotope Geosciences Facility
	Texas A&M University
	College Station, Texas 77843
	United States of America
Data accessibility	With this article
Related research article	E.B. Pinto & N.C. Slowey (2021) Stable isotope evidence for the origins of waters in the Guayas estuary and Gulf of Guayaquil. *Estuarine, Coastal and Shelf Science*, https://doi.org/10.1016/j.ecss.2020.107151*.*

## Value of the Data

• Salinity, δ^18^O, and δ^2^H values for Guayas estuary waters indicate the origins of estuarine waters and environmental processes that affect these waters.
• Researchers interested in estuarine dynamics benefit from knowledge of abundances of water types and movement of water within various parts of the estuary.
• This dataset can be used to support studies of nutrient cycling and pollutant transport, and it can inform the further studies of estuarine circulation and the impacts of seasonal and inter-annual climate variability on the estuary's physical and biological components.

## 1 Data Description

Table 1 presents the geographical coordinates and water depths of each water sample, together with measured values of salinity, δ^18^O, and δ^2^H. For graphic depictions of this data, see Ref. [1].

**Table 1:** Guayas estuary water samples: sample collection information, salinity, δ^18^O, and δ^2^H.

Sample	Latitude	Longitude	Depth	Salinity	δ^18^O_VSMOW_	δ^2^H_VSMOW_
Collected	(°)	(°)	(m)	(PSU)	(‰)	(‰)
11-Dec-13	-3.0611	-80.2197	0	34.8	0.2	0.1
11-Dec-13	-3.0611	-80.2197	5	35.8	0.3	0.2
11-Dec-13	-3.0833	-80.2197	0	34.7	0.2	0.2
11-Dec-13	-3.0833	-80.2197	5	35.9	0.3	0.4
11-Dec-13	-3.0833	-80.2197	10	36.7	0.3	0.3
11-Dec-13	-3.0833	-80.2197	15	36.4	0.5	0.4
11-Dec-13	-3.1067	-80.2197	0	35.6	0.3	0.2
11-Dec-13	-3.1067	-80.2197	5	35.8	0.3	0.3
11-Dec-13	-3.1067	-80.2197	10	36.4	0.3	0.6
11-Dec-13	-3.1300	-80.2197	0	35.8	0.3	0.0
11-Dec-13	-3.1300	-80.2197	5	35.6	0.3	0.3
11-Dec-13	-3.1300	-80.2197	10	35.6	0.2	0.4
11-Dec-13	-3.1517	-80.2197	0	35.8	0.2	0.3
11-Dec-13	-3.1517	-80.2197	5	35.8	-0.1	0.9
11-Dec-13	-3.1750	-80.2197	0	35.6	0.0	0.7
11-Dec-13	-3.1750	-80.2197	5	35.7	-0.1	0.7
11-Dec-13	-3.1978	-80.2197	0	35.3	0.0	0.5
11-Dec-13	-3.1978	-80.2197	5	35.8	0.1	1.0
11-Dec-13	-3.1978	-80.2197	10	35.9	0.0	0.7
11-Dec-13	-3.2200	-80.2197	0	35.8	0.1	0.8
11-Dec-13	-3.2200	-80.2197	5	35.6	0.0	0.7
11-Dec-13	-3.2200	-80.2197	10	36.0	-0.1	1.0
11-Dec-13	-3.2433	-80.2197	0	35.6	0.2	0.3
11-Dec-13	-3.2433	-80.2197	5	35.5	0.0	0.2
11-Dec-13	-3.2647	-80.2197	0	35.9	0.0	0.4
11-Dec-13	-3.2889	-80.2197	0	35.9	0.2	1.1
11-Dec-13	-2.9950	-80.0750	0	35.6	0.0	0.4
11-Dec-13	-2.9950	-80.0750	5	35.5	0.1	0.5
11-Dec-13	-2.9950	-80.0750	10	35.5	0.1	0.5
11-Dec-13	-3.0083	-80.0583	0	35.5	0.2	0.5
11-Dec-13	-3.0083	-80.0583	5	35.2	0.1	0.5
11-Dec-13	-3.0083	-80.0583	10	35.2	0.1	0.3
11-Dec-13	-3.0083	-80.0583	15	35.2	0.0	0.5
11-Dec-13	-3.0233	-80.0417	0	35.4	0.0	0.3
11-Dec-13	-3.0233	-80.0417	5	35.7	0.1	0.7
11-Dec-13	-3.0233	-80.0417	10	35.8	0.1	0.2
11-Dec-13	-3.0233	-80.0417	15	33.9	0.1	0.7
11-Dec-13	-3.0233	-80.0417	20	35.9	0.2	0.7
12-Dec-13	-3.0400	-80.0267	0	36.0	0.2	0.4
12-Dec-13	-3.0400	-80.0267	5	36.0	0.2	0.1
12-Dec-13	-3.0400	-80.0267	10	36.2	0.2	0.6
12-Dec-13	-3.0544	-80.0083	0	36.2	0.2	0.5
12-Dec-13	-3.0544	-80.0083	5	36.0	0.3	0.7
12-Dec-13	-3.0700	-79.9933	0	35.8	0.2	0.3
12-Dec-13	-3.0700	-79.9933	5	35.8	0.3	0.1
12-Dec-13	-3.0867	-79.9767	0	36.0	0.3	0.4
12-Dec-13	-3.0867	-79.9767	5	36.1	0.1	0.7
12-Dec-13	-3.1017	-79.9600	0	35.9	0.2	0.9
12-Dec-13	-3.1017	-79.9600	5	36.0	0.3	0.5
11-Dec-13	-2.7433	-79.8967	0	35.5	0.2	-0.1
11-Dec-13	-2.7433	-79.8967	5	34.4	0.1	-0.3
11-Dec-13	-2.7433	-79.8967	10	34.3	0.1	-0.5
11-Dec-13	-2.7433	-79.8967	15	34.2	0.2	-0.5
11-Dec-13	-2.7433	-79.8811	0	33.1	0.1	-0.8
11-Dec-13	-2.7433	-79.8811	5	33.7	0.0	0.2
11-Dec-13	-2.7433	-79.8644	0	33.1	0.0	-0.8
11-Dec-13	-2.7433	-79.8644	5	33.8	0.1	-0.8
11-Dec-13	-2.7433	-79.8478	0	32.5	-0.1	-0.8
11-Dec-13	-2.7433	-79.8478	5	32.8	-0.1	-0.7
11-Dec-13	-2.7433	-79.8333	0	31.7	-0.2	-1.4
11-Dec-13	-2.7433	-79.8133	0	31.0	-0.1	-1.7
12-Dec-13	-2.6617	-79.8333	0	32.6	0.0	-0.8
12-Dec-13	-2.6617	-79.8333	5	32.7	0.0	-1.2
12-Dec-13	-2.6617	-79.8333	10	32.8	0.1	-0.3
12-Dec-13	-2.6617	-79.8133	0	32.0	0.0	-1.8
12-Dec-13	-2.6617	-79.8133	5	31.9	0.0	-1.6
12-Dec-13	-2.6617	-79.7972	0	30.7	-0.1	-2.7
12-Dec-13	-2.6617	-79.7972	5	30.9	-0.1	-2.3
11-Dec-13	-2.6967	-79.9600	0	33.3	0.2	-0.8
11-Dec-13	-2.6967	-79.9600	5	34.0	0.2	-0.5
11-Dec-13	-2.7000	-79.9631	0	33.0	0.2	-1.0
11-Dec-13	-2.7000	-79.9631	5	33.5	0.0	-0.2
11-Dec-13	-2.7050	-79.9600	0	33.3	-0.1	-1.5
11-Dec-13	-2.6383	-79.9333	0	27.8	-0.7	-4.7
11-Dec-13	-2.6383	-79.9333	5	31.8	-0.2	-1.7
11-Dec-13	-2.6383	-79.9100	0	27.3	-0.7	-4.5
11-Dec-13	-2.6383	-79.9100	5	29.6	-0.7	-5.3
11-Dec-13	-2.6383	-79.8933	0	29.8	-0.7	-5.5
12-Dec-13	-2.5856	-79.8217	0	31.5	-0.3	-2.6
12-Dec-13	-2.5856	-79.8217	5	31.3	-0.2	-1.6
12-Dec-13	-2.5856	-79.8111	0	30.8	-0.3	-2.6
12-Dec-13	-2.5856	-79.8111	5	30.9	-0.2	-2.8
12-Dec-13	-2.5222	-79.8800	0	26.5	-0.6	-5.6
12-Dec-13	-2.5222	-79.8800	5	28.3	-0.3	-4.3
12-Dec-13	-2.5222	-79.8739	0	25.7	-0.6	-5.8
12-Dec-13	-2.5222	-79.8739	5	28.5	-0.4	-4.1
12-Dec-13	-2.5222	-79.8667	0	25.5	-0.7	-6.0
12-Dec-13	-2.5222	-79.8667	5		-0.7	-4.2
12-Dec-13	-2.3578	-79.8483	0	13.7	-2.1	-13.8
12-Dec-13	-2.3578	-79.8483	5	14.9	-2.1	-13.6
12-Dec-13	-2.3578	-79.8378	0	13.3	-2.1	-14.2
12-Dec-13	-2.3578	-79.8378	5	13.5	-2.4	-14.9
12-Dec-13	-2.3578	-79.8300	0	12.0	-2.4	-15.8
12-Dec-13	-2.3578	-79.8300	5	13.3	-2.3	-15.1
12-Dec-13	-2.2467	-79.8756	0	8.2	-3.2	-20.2
12-Dec-13	-2.2467	-79.8756	5	7.5	-3.2	-19.8
12-Dec-13	-2.2467	-79.8756	10	6.6	-3.1	-18.6
12-Dec-13	-2.2467	-79.8600	0	7.0	-3.1	-18.6
12-Dec-13	-2.2467	-79.8600	5	7.0	-3.3	-20.1
12-Dec-13	-2.2467	-79.8381	0	7.0	-3.3	-20.0
12-Dec-13	-2.2467	-79.8317	0	5.2	-3.7	-22.3
10-Dec-13	-2.3567	-80.0150	0	32.1	-0.1	-1.1
10-Dec-13	-2.3567	-80.0150	5	32.2	0.1	-0.6
10-Dec-13	-2.3567	-80.0150	10	32.6	-0.1	-0.6
10-Dec-13	-2.3617	-80.0083	0	32.1	-0.2	-1.1
10-Dec-13	-2.3617	-80.0083	5	30.7	-0.2	-1.1
10-Dec-13	-2.3617	-80.0083	10	30.8	-0.1	-0.7
10-Dec-13	-2.4522	-80.0583	0	32.8	-0.1	-0.6
10-Dec-13	-2.4522	-80.0583	5	32.7	0.1	-0.9
10-Dec-13	-2.4567	-80.0483	0	33.0	0.1	-1.6
10-Dec-13	-2.4567	-80.0483	5	32.7	0.1	-1.0
10-Dec-13	-2.4600	-80.0400	0	32.0	0.0	-1.4
10-Dec-13	-2.4600	-80.0400	5	32.3	0.1	-1.2
10-Dec-13	-2.5250	-80.0978	0	33.5	0.0	-0.6
10-Dec-13	-2.5250	-80.0978	5	33.8	0.1	-0.6
10-Dec-13	-2.5350	-80.0817	0	33.3	0.0	-1.0
10-Dec-13	-2.5350	-80.0817	5	33.0	0.0	-0.7
10-Dec-13	-2.5483	-80.0633	0	33.4	0.0	-0.7
10-Dec-13	-2.5483	-80.0633	5	33.0	0.0	-1.1
10-Dec-13	-2.6500	-80.0889	0	33.0	0.1	-0.7
10-Dec-13	-2.6500	-80.0889	5	33.3	0.0	-1.0
10-Dec-13	-2.6628	-80.0867	0	33.3	0.1	-0.9
10-Dec-13	-2.6628	-80.0867	5	33.0	0.0	-0.5
10-Dec-13	-2.6744	-80.0847	0	33.6	0.0	-0.3
10-Dec-13	-2.6744	-80.0847	5	33.6	-0.1	-0.3
10-Dec-13	-2.6817	-80.2450	0	33.4	0.1	-0.5
10-Dec-13	-2.6817	-80.2450	5	33.7	0.1	-0.1
10-Dec-13	-2.6817	-80.2450	10	33.6	0.1	-0.8
10-Dec-13	-2.6989	-80.2294	0	33.8	0.0	-0.5
10-Dec-13	-2.6989	-80.2294	5	33.6	0.0	-0.3
10-Dec-13	-2.6989	-80.2294	10	33.5	0.0	-0.4
10-Dec-13	-2.6989	-80.2294	15	33.6	-0.2	-0.4
10-Dec-13	-2.6989	-80.2294	20	34.0	-0.2	-0.5
10-Dec-13	-2.7117	-80.2167	0	33.8	-0.1	-0.7
10-Dec-13	-2.7117	-80.2167	5	33.8	-0.2	-0.3
10-Dec-13	-2.7117	-80.2167	10	33.7	-0.1	-0.3
10-Dec-13	-2.7314	-80.2917	0	33.7	-0.1	-0.4
10-Dec-13	-2.7500	-80.2833	0	34.1	-0.1	-0.4
10-Dec-13	-2.7500	-80.2833	5	33.8	-0.1	-0.3
10-Dec-13	-2.7700	-80.2722	0	34.0	0.0	-0.2
10-Dec-13	-2.7700	-80.2722	5	34.0	-0.1	-0.4
10-Dec-13	-2.7700	-80.2722	10	34.0	0.0	-0.1
10-Dec-13	-2.7917	-80.2611	0	33.6	-0.2	0.1
10-Dec-13	-2.7917	-80.2611	5	33.7	-0.2	0.1
10-Dec-13	-2.7917	-80.2611	10	34.1	-0.2	0.1
10-Dec-13	-2.8100	-80.2517	0	33.8	-0.2	-0.4
10-Dec-13	-2.8100	-80.2517	5	33.7	-0.1	-0.1
22-Jan-14	-3.3167	-81.0000	0	33.6	-0.1	-0.3
22-Jan-14	-3.3167	-81.0000	10	34.0	0.0	0.7
22-Jan-14	-3.3167	-81.0000	20	34.6	0.0	1.1
22-Jan-14	-3.3167	-81.0000	30	34.8	0.1	0.9
22-Jan-14	-3.3167	-81.0000	40	34.8	0.1	0.4
22-Jan-14	-3.3167	-81.0000	50	34.9	0.2	1.4
22-Jan-14	-3.3167	-81.0000	75	34.9	0.1	1.4
22-Jan-14	-3.3167	-81.0000	100	35.0	0.1	1.0
22-Jan-14	-2.9667	-81.0000	0	33.7	0.1	-0.4
22-Jan-14	-2.9667	-81.0000	10	33.9	0.2	0.2
22-Jan-14	-2.9667	-81.0000	20	34.7	0.2	0.5
22-Jan-14	-2.9667	-81.0000	30	34.9	0.2	0.1
22-Jan-14	-2.9667	-81.0000	40	34.9	0.2	1.1
22-Jan-14	-2.9667	-81.0000	50	34.9	0.1	1.1
22-Jan-14	-2.9667	-81.0000	75	35.0	0.2	1.3
22-Jan-14	-2.9667	-81.0000	100	35.0	0.2	1.0
22-Jan-14	-2.6167	-81.0000	0	33.6	0.1	-0.5
22-Jan-14	-2.6167	-81.0000	10	33.7	0.1	-0.6
22-Jan-14	-2.6167	-81.0000	20	34.6	0.2	0.4
22-Jan-14	-2.6167	-81.0000	30	34.7	0.2	0.4
22-Jan-14	-2.6167	-81.0000	40	34.9	0.2	0.9
22-Jan-14	-2.6167	-81.0000	50	35.0	0.2	0.8
22-Jan-14	-2.6167	-81.0000	75	35.0	0.1	-0.2
22-Jan-14	-2.6167	-81.0000	100	35.0	0.0	-0.2
23-Jan-14	-2.2833	-81.0000	0	33.5	-0.1	-0.5
23-Jan-14	-2.2833	-81.0000	10	33.8	0.0	-0.2
23-Jan-14	-2.2833	-81.0000	20	34.6	0.0	0.5
23-Jan-14	-2.4333	-80.7833	0	33.5	0.0	-0.3
23-Jan-14	-2.4333	-80.7833	10	33.6	0.0	0.2
24-Jan-14	-2.7333	-80.7833	0	33.5	-0.1	-0.7
24-Jan-14	-2.7333	-80.7833	10	34.0	0.0	-0.2
24-Jan-14	-2.7333	-80.7833	20	34.5	-0.1	0.3
24-Jan-14	-2.7333	-80.7833	30	34.6	0.0	0.4
24-Jan-14	-2.7333	-80.7833	50	34.9	0.3	0.8
22-Jan-14	-3.0333	-80.7833	0	33.6	0.2	-1.2
22-Jan-14	-3.0333	-80.7833	10	34.0	0.2	-0.2
22-Jan-14	-3.0333	-80.7833	20	34.4	0.3	-0.1
22-Jan-14	-3.0333	-80.7833	30	34.7	0.3	0.3
22-Jan-14	-3.0333	-80.7833	40	34.8	0.4	1.0
22-Jan-14	-3.0333	-80.7833	50	34.9	0.3	0.6
22-Jan-14	-3.3167	-80.7833	0	33.6	0.1	-0.3
22-Jan-14	-3.3167	-80.7833	10	33.8	0.4	0.3
22-Jan-14	-3.3167	-80.7833	20	34.4	0.3	0.5
22-Jan-14	-3.3167	-80.7833	30	34.7	0.3	0.7
22-Jan-14	-3.3167	-80.7833	40	34.8	0.3	0.7
22-Jan-14	-3.3167	-80.7833	50	34.9	0.4	0.9
22-Jan-14	-3.3333	-80.5833	0	33.6	0.1	-0.3
22-Jan-14	-3.3333	-80.5833	10	33.7	0.1	-0.3
22-Jan-14	-3.3333	-80.5833	20	34.3	0.4	0.1
22-Jan-14	-3.3333	-80.5833	30	34.6	0.2	0.6
22-Jan-14	-3.3333	-80.5833	40	34.7	0.3	0.6
22-Jan-14	-3.0833	-80.5833	0	33.5	0.2	-0.8
22-Jan-14	-3.0833	-80.5833	10	33.5	0.2	-0.7
22-Jan-14	-3.0833	-80.5833	20	34.0	0.3	0.0
22-Jan-14	-3.0833	-80.5833	30	34.7	0.3	1.0
22-Jan-14	-3.0833	-80.5833	40	34.8	0.1	0.4
25-Jan-14	-2.8333	-80.5833	0	33.5	0.0	-0.7
25-Jan-14	-2.8333	-80.5833	10	33.5	0.0	-0.4
25-Jan-14	-2.6000	-80.5833	0	33.6	0.0	0.1
25-Jan-14	-2.6000	-80.5833	10	33.8	0.0	-0.1
21-Jan-14	-3.1167	-80.3500	0	33.3	0.1	0.1
21-Jan-14	-3.1167	-80.3500	10	33.7	0.1	0.3
21-Jan-14	-3.1167	-80.3500	20	34.3	0.1	0.5
21-Jan-14	-3.1167	-80.3500	30	34.6	0.2	0.7
21-Jan-14	-3.1167	-80.3500	40		0.2	0.8
22-Jan-14	-3.3167	-80.3500	0	33.5	0.0	-0.4
22-Jan-14	-3.3167	-80.3500	10	33.7	0.1	0.0
22-Jan-14	-3.3167	-80.3500	20	34.1	0.2	0.5
18-May-14	-3.0611	-80.2197	0	29.6	-0.7	-3.8
18-May-14	-3.0611	-80.2197	5	30.1	-0.6	-3.0
18-May-14	-3.0611	-80.2197	10	31.6	-0.4	-2.3
18-May-14	-3.0611	-80.2197	15	32.2	-0.3	-1.3
18-May-14	-3.0833	-80.2197	0	28.6	-0.9	-4.8
18-May-14	-3.0833	-80.2197	5	30.5	-0.6	-2.8
18-May-14	-3.0833	-80.2197	10	31.8	-0.4	-1.9
18-May-14	-3.1067	-80.2197	0	29.5	-0.6	-4.0
18-May-14	-3.1067	-80.2197	5	30.8	-0.5	-2.8
18-May-14	-3.1067	-80.2197	10	32.1	-0.2	-1.5
18-May-14	-3.1067	-80.2197	15	32.7	-0.2	-1.3
18-May-14	-3.1300	-80.2197	0	30.1	-0.6	-3.5
18-May-14	-3.1300	-80.2197	5	31.2	-0.5	-2.5
18-May-14	-3.1300	-80.2197	10	32.1	-0.4	-1.7
18-May-14	-3.1517	-80.2197	0	29.6	-0.7	-4.1
18-May-14	-3.1517	-80.2197	5	31.2	-0.3	-3.5
18-May-14	-3.1517	-80.2197	10	32.7	-0.2	-1.8
18-May-14	-3.1750	-80.2197	0	28.6	-0.9	-6.3
18-May-14	-3.1750	-80.2197	5	30.3	-0.6	-3.7
18-May-14	-3.1750	-80.2197	10	32.8	-0.4	-1.8
18-May-14	-3.1978	-80.2197	0	29.6	-0.8	-4.4
18-May-14	-3.1978	-80.2197	5	31.1	-0.7	-2.8
18-May-14	-3.1978	-80.2197	10	33.2	-0.2	-0.7
18-May-14	-3.2200	-80.2197	0	29.7	-0.8	-4.2
18-May-14	-3.2200	-80.2197	5	30.0	-0.7	-4.0
18-May-14	-3.2200	-80.2197	10	33.3	-0.1	-0.4
18-May-14	-3.2433	-80.2197	0	30.2	-0.5	-4.0
18-May-14	-3.2433	-80.2197	5	30.6	-0.6	-3.4
18-May-14	-3.2433	-80.2197	10	32.5	-0.4	-1.1
18-May-14	-3.2647	-80.2197	0	30.2	-0.7	-3.7
18-May-14	-3.2647	-80.2197	5	31.4	-0.6	-2.8
18-May-14	-3.2647	-80.2197	10	32.9	-0.3	-1.0
18-May-14	-3.2647	-80.2197	15	33.5	-0.1	-0.3
18-May-14	-3.2889	-80.2197	0	31.3	-0.5	-2.5
18-May-14	-3.2889	-80.2197	5	31.3	-0.6	-2.7
18-May-14	-2.9950	-80.0750	0	29.9	-0.5	-3.5
18-May-14	-2.9950	-80.0750	5	30.2	-0.5	-3.4
18-May-14	-2.9950	-80.0750	10	30.8	-0.5	-2.6
18-May-14	-2.9950	-80.0750	15	31.7	-0.3	-2.0
18-May-14	-3.0083	-80.0583	0	29.7	-0.7	-3.8
18-May-14	-3.0083	-80.0583	5	29.6	-0.7	-4.0
18-May-14	-3.0083	-80.0583	10	29.8	-0.7	-3.7
18-May-14	-3.0083	-80.0583	15	31.2	-0.4	-2.2
18-May-14	-3.0083	-80.0583	20	31.4	-0.4	-2.3
18-May-14	-3.0233	-80.0417	0	28.2	-0.9	-5.3
18-May-14	-3.0233	-80.0417	5	28.8	-0.8	-4.8
18-May-14	-3.0233	-80.0417	10	29.5	-0.7	-4.2
18-May-14	-3.0400	-80.0267	0	28.3	-0.9	-5.4
18-May-14	-3.0400	-80.0267	5	28.9	-0.7	-4.9
18-May-14	-3.0544	-80.0083	0	25.1	-1.4	-8.3
18-May-14	-3.0544	-80.0083	5	25.9	-1.1	-9.2
18-May-14	-3.0700	-79.9933	0	26.0	-0.7	-10.0
18-May-14	-3.0700	-79.9933	5	27.0	-0.3	-9.7
18-May-14	-3.0867	-79.9767	0	26.3	-0.3	-10.9
18-May-14	-3.0867	-79.9767	5	26.5	-0.1	-11.1
18-May-14	-3.1017	-79.9600	0	26.2	-0.2	-11.0
18-May-14	-3.1017	-79.9600	5	26.3	-0.3	-10.8
19-May-14	-2.7433	-79.8967	0	18.9	-2.6	-15.9
19-May-14	-2.7433	-79.8967	5	19.9	-2.6	-15.3
19-May-14	-2.7433	-79.8967	10	22.2	-2.0	-11.3
19-May-14	-2.7433	-79.8967	15	22.9	-2.3	-13.2
19-May-14	-2.7433	-79.8811	0	20.2	-2.4	-14.3
19-May-14	-2.7433	-79.8811	5	21.8	-2.0	-12.6
19-May-14	-2.7433	-79.8811	10	22.5	-2.0	-11.5
19-May-14	-2.7433	-79.8644	0	18.0	-3.0	-17.2
19-May-14	-2.7433	-79.8644	5	18.8	-2.8	-16.0
19-May-14	-2.7433	-79.8644	10	20.4	-2.5	-14.1
19-May-14	-2.7433	-79.8478	0	18.7	-2.4	-14.5
19-May-14	-2.7433	-79.8478	5	19.8	-2.2	-13.4
19-May-14	-2.7433	-79.8333	0	19.2	-2.4	-14.3
19-May-14	-2.7433	-79.8333	10	20.4	-2.2	-12.7
19-May-14	-2.7433	-79.8133	0	13.7	-3.3	-20.8
19-May-14	-2.7433	-79.8133	5	15.5	-2.9	-18.3
19-May-14	-2.6617	-79.8333	0	12.6	-3.6	-22.3
19-May-14	-2.6617	-79.8333	5	14.8	-3.1	-19.7
19-May-14	-2.6617	-79.8333	10	16.4	-2.8	-17.8
19-May-14	-2.6617	-79.8133	0	12.7	-3.7	-22.4
19-May-14	-2.6617	-79.8133	5	13.1	-3.5	-21.8
19-May-14	-2.6617	-79.7972	0	8.1	-4.4	-28.2
19-May-14	-2.6617	-79.7972	5	12.4	-3.6	-22.7
17-May-14	-2.6967	-79.9600	0	18.9	-2.3	-14.3
17-May-14	-2.6967	-79.9600	5	20.0	-2.3	-12.9
17-May-14	-2.7000	-79.9631	0	20.0	-2.1	-13.0
17-May-14	-2.7000	-79.9631	5	19.9	-2.1	-13.2
17-May-14	-2.7050	-79.9600	0	19.3	-2.2	-13.9
20-May-14	-2.6383	-79.9333	0	15.5	-3.0	-19.2
20-May-14	-2.6383	-79.9333	5	17.5	-2.6	-16.6
20-May-14	-2.6383	-79.9100	0	15.6	-3.0	-18.8
20-May-14	-2.6383	-79.9100	5	17.4	-2.6	-16.8
20-May-14	-2.6383	-79.8933	0	14.1	-3.2	-20.8
20-May-14	-2.6383	-79.8933	5	15.7	-2.9	-19.3
19-May-14	-2.5856	-79.8217	0	8.9	-4.3	-27.4
19-May-14	-2.5856	-79.8217	5	10.1	-4.1	-25.9
19-May-14	-2.5856	-79.8111	0	8.9	-4.3	-27.3
19-May-14	-2.5856	-79.8111	5	10.1	-4.0	-26.0
20-May-14	-2.5222	-79.8800	0	6.1	-4.9	-31.3
20-May-14	-2.5222	-79.8800	5	6.4	-4.9	-30.8
20-May-14	-2.5222	-79.8739	0	6.2	-4.9	-31.0
20-May-14	-2.5222	-79.8739	5	6.9	-4.8	-30.0
20-May-14	-2.5222	-79.8667	0	5.3	-5.1	-32.1
20-May-14	-2.5222	-79.8667	5	7.4	-4.7	-29.5
19-May-14	-2.3578	-79.8483	0	0.2	-6.0	-38.0
19-May-14	-2.3578	-79.8483	5	0.2	-6.0	-38.8
20-May-14	-2.3578	-79.8378	0	0.2	-6.0	-38.6
20-May-14	-2.3578	-79.8378	5	0.2	-5.9	-38.8
20-May-14	-2.3578	-79.8300	0	0.2	-6.0	-38.6
20-May-14	-2.3578	-79.8300	5	0.2	-6.1	-38.7
19-May-14	-2.2467	-79.8756	0	0.2	-6.0	-38.0
19-May-14	-2.2467	-79.8756	5	0.2	-5.9	-38.3
20-May-14	-2.2467	-79.8600	0	0.1	-5.8	-36.5
20-May-14	-2.2467	-79.8600	5	0.1	-5.9	-36.8
20-May-14	-2.2467	-79.8381	0	0.1	-6.1	-38.9
20-May-14	-2.2467	-79.8317	0	0.1	-6.0	-39.0
16-May-14	-2.3567	-80.0150	0	19.7	-1.7	-9.9
16-May-14	-2.3567	-80.0150	5	19.6	-1.6	-10.0
16-May-14	-2.3567	-80.0150	10	19.6	-1.6	-9.8
16-May-14	-2.3617	-80.0083	0	19.6	-1.6	-9.9
16-May-14	-2.3617	-80.0083	5	19.7	-1.6	-9.6
16-May-14	-2.3617	-80.0083	10	19.8	-1.7	-9.5
16-May-14	-2.4522	-80.0583	0	20.4	-1.6	-9.2
16-May-14	-2.4522	-80.0583	5	20.4	-1.4	-9.4
16-May-14	-2.4567	-80.0483	0	20.2	-1.5	-9.3
16-May-14	-2.4567	-80.0483	5	20.1	-1.5	-9.2
16-May-14	-2.4600	-80.0400	0	19.0	-1.8	-10.3
16-May-14	-2.4600	-80.0400	5	19.3	-1.7	-10.3
16-May-14	-2.5250	-80.0978	0	20.3	-1.6	-9.4
16-May-14	-2.5250	-80.0978	5	20.4	-1.6	-9.5
16-May-14	-2.5350	-80.0817	0	20.6	-1.5	-9.3
16-May-14	-2.5350	-80.0817	5	20.5	-1.7	-9.2
16-May-14	-2.5483	-80.0633	0	14.0	-3.0	-18.1
16-May-14	-2.5483	-80.0633	5	17.7	-2.3	-14.2
17-May-14	-2.6500	-80.0889	0	20.8	-1.8	-10.8
17-May-14	-2.6500	-80.0889	5	20.8	-1.8	-10.6
17-May-14	-2.6628	-80.0867	0	19.8	-2.3	-12.7
17-May-14	-2.6628	-80.0867	5	19.7	-2.3	-12.6
17-May-14	-2.6744	-80.0847	0	19.0	-2.4	-13.7
16-May-14	-2.6817	-80.2450	0	23.8	-1.4	-8.1
16-May-14	-2.6817	-80.2450	5	23.8	-1.4	-8.0
16-May-14	-2.6817	-80.2450	10	23.8	-1.4	-7.9
16-May-14	-2.6989	-80.2294	0	21.3	-1.9	-10.4
16-May-14	-2.6989	-80.2294	5	21.7	-1.8	-10.0
16-May-14	-2.6989	-80.2294	10	23.2	-1.5	-8.7
16-May-14	-2.6989	-80.2294	15	23.5	-1.5	-8.3
16-May-14	-2.6989	-80.2294	20	23.5	-1.5	-9.0
17-May-14	-2.7117	-80.2167	0	17.4	-2.2	-13.5
17-May-14	-2.7117	-80.2167	5	20.9	-1.9	-11.5
17-May-14	-2.7117	-80.2167	10	21.5	-1.9	-11.3
17-May-14	-2.7500	-80.2833	0	26.5	-0.1	-9.6
17-May-14	-2.7500	-80.2833	5	26.7	-0.2	-9.3
17-May-14	-2.7700	-80.2722	0	27.6	-0.1	-8.4
17-May-14	-2.7700	-80.2722	5	27.5	-0.1	-8.5
17-May-14	-2.7700	-80.2722	10	27.7	0.0	-8.4
17-May-14	-2.7917	-80.2611	0	26.1	-0.3	-9.8
17-May-14	-2.7917	-80.2611	5	26.5	-0.2	-9.3
17-May-14	-2.7917	-80.2611	10	27.1	-0.6	-7.3
17-May-14	-2.8100	-80.2517	0	26.6	-1.3	-5.9
17-May-14	-2.8100	-80.2517	5	26.9	-1.2	-5.9

## 2 Methods

The water samples were collected using Van Dorn bottles lowered into the water with lines, typically from 9.5-m-long survey boats and at a few sites from bridges. Geographical coordinates of the boats were determined using C–Nav GPS systems. The Van Dorn bottles were triggered when they reached specified depths and then brought back aboard the boats, where aliquots of water for salinity and stable isotope analyses were promptly put in 250 ml glass bottles and 20 ml glass vials, respectively, and sealed using conic plastic caps and electrical tape. These bottles were kept under refrigerated conditions prior to being analyzed.

The 250 ml bottles with Guayas estuary water samples were taken to the Department of Chemical Oceanography at the Instituto Oceanográfico de la Armada (INOCAR) in Guayaquil, Ecuador, where a HACH HQ14d meter with conductivity probe was used to determine their salinities. A water sample's conductivity depends on the amount of dissolved salts in it and the temperature at which conductivity is measured. The response of the conductivity probe was calibrated by inserting it into solutions of known salinity and temperature, then the probe was inserted into aliquots of water from the Guayas estuary samples and their conductivity and temperature were measured. Based upon the established probe response, the salinity of each water sample was calculated from its measured conductivity and temperature. Standard procedures for instrument calibration and the direct measurement method were followed (for example, see Ref. [3]).

To assess the consistency of salinity measurements, aliquots of a standard solution were periodically analyzed with the Guayas estuary samples. The estimated precision of an individual salinity analysis is ±0.1 on the PSS-78 practical salinity scale, as indicated by the standard deviation of the replicate analyses of standards. Replicate analyses of the salinities of several randomly selected Guayas estuary samples were also made. In each instance, the difference between each pair of measured values was smaller than the estimated precision of the individual measurements, so a single value for each sample is presented in Table 1.

The salinity values of discrete water samples determined in the laboratory were compared to *in situ* profiles of salinity obtained with a SeaBird CTD at the sites where the water samples were collected. The water sample values agreed well with the *in situ* profile values, indicating the Van Dorn bottles triggered at the intended water depths and the samples were not affected by handling prior to being analyzed.

The 20 ml vials with Guayas estuary water samples were taken to the Stable Isotope Geosciences Facility at Texas A&M University in College Station, Texas, USA. Their ^18^O/^16^O and ^2^H/^1^H ratios where measured using a Picarro L2120i cavity ring-down spectroscopy (CRDS) analyzer with a vaporization module. The near-infrared absorption spectrum of water vapor molecules depends upon their oxygen and hydrogen isotopic compositions. An auto-sampler delivered an aliquot of liquid water to the CRDS system, where it was converted to vapor and its near-infrared absorption spectrum was determined at a constant temperature (e.g., method of Gupta et al. [2]).

Isotope measurements of Guayas estuary water samples were calibrated to the SIGF2013 and JGULF internal laboratory standards, which in turn, were calibrated using VSMOW, GISP, and SLAP standards. The oxygen and hydrogen isotopic compositions of the Guayas estuary water samples are expressed in δ notation relative to VSMOW (Table 1). Typically, two standards were analyzed with every seven Guayas estuary water samples. Analytical precisions of individual δ^18^O and δ^2^H analyses are ±0.1‰ and ±1.0‰, respectively, as estimated from the standard deviations of values obtained from replicate analyses of the internal laboratory standards.

## Ethics Statement

Both authors contributed substantially to the production of this dataset, and it did not involve any human subjects or animal experiments.

## CRediT Author Statement

**Niall C. Slowey:** Conceptualization, Methodology, Resources, Writing - original draft; **Edwin B. Pinto:** Conceptualization, Methodology, Resources, Investigation, Writing - review & editing.

## Declaration of Competing Interest

The authors declare that they have no competing financial interests or personal relationships that have, or could be perceived to have, influenced the work reported in this article.

## Appendix Supplementary materials

Image, application 1
