# Supplementary material for: Salinity and stable isotope dataset for Guayas estuary waters
Source: Data Brief. 2021 Jan 19;35:106776. doi: 10.1016/j.dib.2021.106776 (PMC7844431; doi:10.1016/j.dib.2021.106776)
Supplement: Supplementary file 1 [file mmc1.docx]

**Article Title**

Salinity and stable isotope dataset for Guayas estuary waters

**Authors**

Niall C. Slowey^1^ and Edwin B. Pinto^1,2^

**Affiliations**

1. Department of Oceanography, Texas A&M University, College Station, Texas 77843 U.S.A.

2. Instituto Oceanográfico de la Armada (INOCAR), Av. 25 de Julio, Guayaquil, Ecuador

**Corresponding author(s)**

Edwin Pinto (epinto@armada.mil.ec) and Niall Slowey ([slowey@ocean.tamu.edu](mailto:slowey@ocean.tamu.edu))

**Abstract**

The Guayas estuary is an ecologically and economically vital, large estuarine system located on the western coast of South America. A suite of ~400 water samples was collected and analyzed to enable investigation of the sources of water types within this estuary, as well as environmental processes active within various portions of it. These samples were obtained at sites distributed across the major areas of the inner and outer portions of the estuary at the ends of consecutive dry (December/January) and rainy (May) seasons. At each site, Van Dorn bottles were lowered into the water from a boat (or bridge) and then triggered when they reached specified depths. When the Van Dorn bottles were brought back aboard the boat, aliquots of water from them were promptly sealed in glass containers for later analysis. They were transported to onshore laboratories where their salinities, and their oxygen (δ^18^O) and hydrogen (δ^2^H) isotopic compositions were measured. Established analytical procedures and standards were employed to obtain a robust set of resultant data. The samples, analyses, and data presented here support the associated research article, “Stable isotope evidence for the origins of waters in the Guayas estuary and Gulf of Guayaquil” [1], to which readers are referred for interpretation.

**Keywords**

Guayas estuary; oxygen isotope and hydrogen isotope ratios; salinity; water properties

**Specifications Table**

| **Subject** | Earth and Planetary Sciences |
| --- | --- |
| **Specific subject area** | Oceanography |
| **Type of data** | Table |
| **How data were acquired** | Water samples were collected in the field. Salinities were determined using a HACH HQ14d conductivity meter; oxygen and hydrogen isotope ratios were determined using a Picarro L2120i cavity ring-down spectroscopy (CRDS) analyzer. |
| **Data format** | Raw and analyzed data |
| **Parameters for data collection** | Water sample collection date, latitude (°), longitude (°), depth (m), salinity (PSU), oxygen isotope ratio (‰), and hydrogen isotope ratio (‰) |
| **Description of data collection** | Sampling devices were deployed from boats to collect waters at sites and water depths distributed across the Guayas estuary. Their salinities were determined from measured conductivity (±0.1 precision on PSS-78 practical salinity scale) at INOCAR, and their stable isotope ratios of oxygen (δ^18^O) and hydrogen (δ^2^H) were measured at Texas A&M University using a CRDS analyzer at (precisions of individual δ^18^O and δ^2^H analyses relative to VSMOW are ±0.1‰ and ±1.0‰, respectively). |
| **Data source location** | Water samples were collected in the Guayas estuary of Ecuador (see Table 1 for specific coordinates). The samples were analyzed at these institutions:  1) Marine Chemistry Laboratory  Instituto Oceanográfico de la Armada (INOCAR)  Av. 25 de Julio  Guayaquil  Ecuador  2) Stable Isotope Geosciences Facility  Texas A&M University  College Station, Texas 77843  United States of America |
| **Data accessibility** | With this article |
| **Related research article** | E.B. Pinto & N.C. Slowey (2021) Stable isotope evidence for the origins of waters in the Guayas estuary and Gulf of Guayaquil. *Estuarine, Coastal and Shelf Science*, doi: https:// doi.org/10.1016/j.ecss.2020.107151*.* |

**Value of the Data**

- Salinity, δ^18^O, and δ^2^H values for Guayas estuary waters indicate the origins of estuarine waters and environmental processes that affect these waters.
- Researchers interested in estuarine dynamics benefit from knowledge of abundances of water types and movement of water within various parts of the estuary.
- This dataset can be used to support studies of nutrient cycling and pollutant transport, and it can inform the further studies of estuarine circulation and the impacts of seasonal and inter-annual climate variability on the estuary’s physical and biological components.

**Data Description**

Table 1 presents the geographical coordinates and water depths of each water sample, together with measured values of salinity, δ^18^O, and δ^2^H. For graphic depictions of this data, see reference [1].

**Table 1.** Guayas estuary water samples: sample collection information, salinity, δ^18^O, and δ^2^H

Sample Latitude Longitude Depth Salinity δ^18^O_VSMOW_ δ^2^H_VSMOW_

Collected (°) (°) (m) (PSU) (‰) (‰)

11-Dec-13 -3.0611 -80.2197 0 34.8 0.2 0.1

11-Dec-13 -3.0611 -80.2197 5 35.8 0.3 0.2

11-Dec-13 -3.0833 -80.2197 0 34.7 0.2 0.2

11-Dec-13 -3.0833 -80.2197 5 35.9 0.3 0.4

11-Dec-13 -3.0833 -80.2197 10 36.7 0.3 0.3

11-Dec-13 -3.0833 -80.2197 15 36.4 0.5 0.4

11-Dec-13 -3.1067 -80.2197 0 35.6 0.3 0.2

11-Dec-13 -3.1067 -80.2197 5 35.8 0.3 0.3

11-Dec-13 -3.1067 -80.2197 10 36.4 0.3 0.6

11-Dec-13 -3.1300 -80.2197 0 35.8 0.3 0.0

11-Dec-13 -3.1300 -80.2197 5 35.6 0.3 0.3

11-Dec-13 -3.1300 -80.2197 10 35.6 0.2 0.4

11-Dec-13 -3.1517 -80.2197 0 35.8 0.2 0.3

11-Dec-13 -3.1517 -80.2197 5 35.8 -0.1 0.9

11-Dec-13 -3.1750 -80.2197 0 35.6 0.0 0.7

11-Dec-13 -3.1750 -80.2197 5 35.7 -0.1 0.7

11-Dec-13 -3.1978 -80.2197 0 35.3 0.0 0.5

11-Dec-13 -3.1978 -80.2197 5 35.8 0.1 1.0

11-Dec-13 -3.1978 -80.2197 10 35.9 0.0 0.7

11-Dec-13 -3.2200 -80.2197 0 35.8 0.1 0.8

11-Dec-13 -3.2200 -80.2197 5 35.6 0.0 0.7

11-Dec-13 -3.2200 -80.2197 10 36.0 -0.1 1.0

11-Dec-13 -3.2433 -80.2197 0 35.6 0.2 0.3

11-Dec-13 -3.2433 -80.2197 5 35.5 0.0 0.2

11-Dec-13 -3.2647 -80.2197 0 35.9 0.0 0.4

11-Dec-13 -3.2889 -80.2197 0 35.9 0.2 1.1

11-Dec-13 -2.9950 -80.0750 0 35.6 0.0 0.4

11-Dec-13 -2.9950 -80.0750 5 35.5 0.1 0.5

11-Dec-13 -2.9950 -80.0750 10 35.5 0.1 0.5

11-Dec-13 -3.0083 -80.0583 0 35.5 0.2 0.5

11-Dec-13 -3.0083 -80.0583 5 35.2 0.1 0.5

11-Dec-13 -3.0083 -80.0583 10 35.2 0.1 0.3

11-Dec-13 -3.0083 -80.0583 15 35.2 0.0 0.5

11-Dec-13 -3.0233 -80.0417 0 35.4 0.0 0.3

11-Dec-13 -3.0233 -80.0417 5 35.7 0.1 0.7

11-Dec-13 -3.0233 -80.0417 10 35.8 0.1 0.2

11-Dec-13 -3.0233 -80.0417 15 33.9 0.1 0.7

11-Dec-13 -3.0233 -80.0417 20 35.9 0.2 0.7

12-Dec-13 -3.0400 -80.0267 0 36.0 0.2 0.4

12-Dec-13 -3.0400 -80.0267 5 36.0 0.2 0.1

12-Dec-13 -3.0400 -80.0267 10 36.2 0.2 0.6

12-Dec-13 -3.0544 -80.0083 0 36.2 0.2 0.5

12-Dec-13 -3.0544 -80.0083 5 36.0 0.3 0.7

12-Dec-13 -3.0700 -79.9933 0 35.8 0.2 0.3

12-Dec-13 -3.0700 -79.9933 5 35.8 0.3 0.1

12-Dec-13 -3.0867 -79.9767 0 36.0 0.3 0.4

12-Dec-13 -3.0867 -79.9767 5 36.1 0.1 0.7

12-Dec-13 -3.1017 -79.9600 0 35.9 0.2 0.9

12-Dec-13 -3.1017 -79.9600 5 36.0 0.3 0.5

11-Dec-13 -2.7433 -79.8967 0 35.5 0.2 -0.1

11-Dec-13 -2.7433 -79.8967 5 34.4 0.1 -0.3

11-Dec-13 -2.7433 -79.8967 10 34.3 0.1 -0.5

11-Dec-13 -2.7433 -79.8967 15 34.2 0.2 -0.5

11-Dec-13 -2.7433 -79.8811 0 33.1 0.1 -0.8

11-Dec-13 -2.7433 -79.8811 5 33.7 0.0 0.2

11-Dec-13 -2.7433 -79.8644 0 33.1 0.0 -0.8

11-Dec-13 -2.7433 -79.8644 5 33.8 0.1 -0.8

11-Dec-13 -2.7433 -79.8478 0 32.5 -0.1 -0.8

11-Dec-13 -2.7433 -79.8478 5 32.8 -0.1 -0.7

11-Dec-13 -2.7433 -79.8333 0 31.7 -0.2 -1.4

11-Dec-13 -2.7433 -79.8133 0 31.0 -0.1 -1.7

12-Dec-13 -2.6617 -79.8333 0 32.6 0.0 -0.8

12-Dec-13 -2.6617 -79.8333 5 32.7 0.0 -1.2

12-Dec-13 -2.6617 -79.8333 10 32.8 0.1 -0.3

12-Dec-13 -2.6617 -79.8133 0 32.0 0.0 -1.8

12-Dec-13 -2.6617 -79.8133 5 31.9 0.0 -1.6

12-Dec-13 -2.6617 -79.7972 0 30.7 -0.1 -2.7

12-Dec-13 -2.6617 -79.7972 5 30.9 -0.1 -2.3

11-Dec-13 -2.6967 -79.9600 0 33.3 0.2 -0.8

11-Dec-13 -2.6967 -79.9600 5 34.0 0.2 -0.5

11-Dec-13 -2.7000 -79.9631 0 33.0 0.2 -1.0

11-Dec-13 -2.7000 -79.9631 5 33.5 0.0 -0.2

11-Dec-13 -2.7050 -79.9600 0 33.3 -0.1 -1.5

11-Dec-13 -2.6383 -79.9333 0 27.8 -0.7 -4.7

11-Dec-13 -2.6383 -79.9333 5 31.8 -0.2 -1.7

11-Dec-13 -2.6383 -79.9100 0 27.3 -0.7 -4.5

11-Dec-13 -2.6383 -79.9100 5 29.6 -0.7 -5.3

11-Dec-13 -2.6383 -79.8933 0 29.8 -0.7 -5.5

12-Dec-13 -2.5856 -79.8217 0 31.5 -0.3 -2.6

12-Dec-13 -2.5856 -79.8217 5 31.3 -0.2 -1.6

12-Dec-13 -2.5856 -79.8111 0 30.8 -0.3 -2.6

12-Dec-13 -2.5856 -79.8111 5 30.9 -0.2 -2.8

12-Dec-13 -2.5222 -79.8800 0 26.5 -0.6 -5.6

12-Dec-13 -2.5222 -79.8800 5 28.3 -0.3 -4.3

12-Dec-13 -2.5222 -79.8739 0 25.7 -0.6 -5.8

12-Dec-13 -2.5222 -79.8739 5 28.5 -0.4 -4.1

12-Dec-13 -2.5222 -79.8667 0 25.5 -0.7 -6.0

12-Dec-13 -2.5222 -79.8667 5 -0.7 -4.2

12-Dec-13 -2.3578 -79.8483 0 13.7 -2.1 -13.8

12-Dec-13 -2.3578 -79.8483 5 14.9 -2.1 -13.6

12-Dec-13 -2.3578 -79.8378 0 13.3 -2.1 -14.2

12-Dec-13 -2.3578 -79.8378 5 13.5 -2.4 -14.9

12-Dec-13 -2.3578 -79.8300 0 12.0 -2.4 -15.8

12-Dec-13 -2.3578 -79.8300 5 13.3 -2.3 -15.1

12-Dec-13 -2.2467 -79.8756 0 8.2 -3.2 -20.2

12-Dec-13 -2.2467 -79.8756 5 7.5 -3.2 -19.8

12-Dec-13 -2.2467 -79.8756 10 6.6 -3.1 -18.6

12-Dec-13 -2.2467 -79.8600 0 7.0 -3.1 -18.6

12-Dec-13 -2.2467 -79.8600 5 7.0 -3.3 -20.1

12-Dec-13 -2.2467 -79.8381 0 7.0 -3.3 -20.0

12-Dec-13 -2.2467 -79.8317 0 5.2 -3.7 -22.3

10-Dec-13 -2.3567 -80.0150 0 32.1 -0.1 -1.1

10-Dec-13 -2.3567 -80.0150 5 32.2 0.1 -0.6

10-Dec-13 -2.3567 -80.0150 10 32.6 -0.1 -0.6

10-Dec-13 -2.3617 -80.0083 0 32.1 -0.2 -1.1

10-Dec-13 -2.3617 -80.0083 5 30.7 -0.2 -1.1

10-Dec-13 -2.3617 -80.0083 10 30.8 -0.1 -0.7

10-Dec-13 -2.4522 -80.0583 0 32.8 -0.1 -0.6

10-Dec-13 -2.4522 -80.0583 5 32.7 0.1 -0.9

10-Dec-13 -2.4567 -80.0483 0 33.0 0.1 -1.6

10-Dec-13 -2.4567 -80.0483 5 32.7 0.1 -1.0

10-Dec-13 -2.4600 -80.0400 0 32.0 0.0 -1.4

10-Dec-13 -2.4600 -80.0400 5 32.3 0.1 -1.2

10-Dec-13 -2.5250 -80.0978 0 33.5 0.0 -0.6

10-Dec-13 -2.5250 -80.0978 5 33.8 0.1 -0.6

10-Dec-13 -2.5350 -80.0817 0 33.3 0.0 -1.0

10-Dec-13 -2.5350 -80.0817 5 33.0 0.0 -0.7

10-Dec-13 -2.5483 -80.0633 0 33.4 0.0 -0.7

10-Dec-13 -2.5483 -80.0633 5 33.0 0.0 -1.1

10-Dec-13 -2.6500 -80.0889 0 33.0 0.1 -0.7

10-Dec-13 -2.6500 -80.0889 5 33.3 0.0 -1.0

10-Dec-13 -2.6628 -80.0867 0 33.3 0.1 -0.9

10-Dec-13 -2.6628 -80.0867 5 33.0 0.0 -0.5

10-Dec-13 -2.6744 -80.0847 0 33.6 0.0 -0.3

10-Dec-13 -2.6744 -80.0847 5 33.6 -0.1 -0.3

10-Dec-13 -2.6817 -80.2450 0 33.4 0.1 -0.5

10-Dec-13 -2.6817 -80.2450 5 33.7 0.1 -0.1

10-Dec-13 -2.6817 -80.2450 10 33.6 0.1 -0.8

10-Dec-13 -2.6989 -80.2294 0 33.8 0.0 -0.5

10-Dec-13 -2.6989 -80.2294 5 33.6 0.0 -0.3

10-Dec-13 -2.6989 -80.2294 10 33.5 0.0 -0.4

10-Dec-13 -2.6989 -80.2294 15 33.6 -0.2 -0.4

10-Dec-13 -2.6989 -80.2294 20 34.0 -0.2 -0.5

10-Dec-13 -2.7117 -80.2167 0 33.8 -0.1 -0.7

10-Dec-13 -2.7117 -80.2167 5 33.8 -0.2 -0.3

10-Dec-13 -2.7117 -80.2167 10 33.7 -0.1 -0.3

10-Dec-13 -2.7314 -80.2917 0 33.7 -0.1 -0.4

10-Dec-13 -2.7500 -80.2833 0 34.1 -0.1 -0.4

10-Dec-13 -2.7500 -80.2833 5 33.8 -0.1 -0.3

10-Dec-13 -2.7700 -80.2722 0 34.0 0.0 -0.2

10-Dec-13 -2.7700 -80.2722 5 34.0 -0.1 -0.4

10-Dec-13 -2.7700 -80.2722 10 34.0 0.0 -0.1

10-Dec-13 -2.7917 -80.2611 0 33.6 -0.2 0.1

10-Dec-13 -2.7917 -80.2611 5 33.7 -0.2 0.1

10-Dec-13 -2.7917 -80.2611 10 34.1 -0.2 0.1

10-Dec-13 -2.8100 -80.2517 0 33.8 -0.2 -0.4

10-Dec-13 -2.8100 -80.2517 5 33.7 -0.1 -0.1

22-Jan-14 -3.3167 -81.0000 0 33.6 -0.1 -0.3

22-Jan-14 -3.3167 -81.0000 10 34.0 0.0 0.7

22-Jan-14 -3.3167 -81.0000 20 34.6 0.0 1.1

22-Jan-14 -3.3167 -81.0000 30 34.8 0.1 0.9

22-Jan-14 -3.3167 -81.0000 40 34.8 0.1 0.4

22-Jan-14 -3.3167 -81.0000 50 34.9 0.2 1.4

22-Jan-14 -3.3167 -81.0000 75 34.9 0.1 1.4

22-Jan-14 -3.3167 -81.0000 100 35.0 0.1 1.0

22-Jan-14 -2.9667 -81.0000 0 33.7 0.1 -0.4

22-Jan-14 -2.9667 -81.0000 10 33.9 0.2 0.2

22-Jan-14 -2.9667 -81.0000 20 34.7 0.2 0.5

22-Jan-14 -2.9667 -81.0000 30 34.9 0.2 0.1

22-Jan-14 -2.9667 -81.0000 40 34.9 0.2 1.1

22-Jan-14 -2.9667 -81.0000 50 34.9 0.1 1.1

22-Jan-14 -2.9667 -81.0000 75 35.0 0.2 1.3

22-Jan-14 -2.9667 -81.0000 100 35.0 0.2 1.0

22-Jan-14 -2.6167 -81.0000 0 33.6 0.1 -0.5

22-Jan-14 -2.6167 -81.0000 10 33.7 0.1 -0.6

22-Jan-14 -2.6167 -81.0000 20 34.6 0.2 0.4

22-Jan-14 -2.6167 -81.0000 30 34.7 0.2 0.4

22-Jan-14 -2.6167 -81.0000 40 34.9 0.2 0.9

22-Jan-14 -2.6167 -81.0000 50 35.0 0.2 0.8

22-Jan-14 -2.6167 -81.0000 75 35.0 0.1 -0.2

22-Jan-14 -2.6167 -81.0000 100 35.0 0.0 -0.2

23-Jan-14 -2.2833 -81.0000 0 33.5 -0.1 -0.5

23-Jan-14 -2.2833 -81.0000 10 33.8 0.0 -0.2

23-Jan-14 -2.2833 -81.0000 20 34.6 0.0 0.5

23-Jan-14 -2.4333 -80.7833 0 33.5 0.0 -0.3

23-Jan-14 -2.4333 -80.7833 10 33.6 0.0 0.2

24-Jan-14 -2.7333 -80.7833 0 33.5 -0.1 -0.7

24-Jan-14 -2.7333 -80.7833 10 34.0 0.0 -0.2

24-Jan-14 -2.7333 -80.7833 20 34.5 -0.1 0.3

24-Jan-14 -2.7333 -80.7833 30 34.6 0.0 0.4

24-Jan-14 -2.7333 -80.7833 50 34.9 0.3 0.8

22-Jan-14 -3.0333 -80.7833 0 33.6 0.2 -1.2

22-Jan-14 -3.0333 -80.7833 10 34.0 0.2 -0.2

22-Jan-14 -3.0333 -80.7833 20 34.4 0.3 -0.1

22-Jan-14 -3.0333 -80.7833 30 34.7 0.3 0.3

22-Jan-14 -3.0333 -80.7833 40 34.8 0.4 1.0

22-Jan-14 -3.0333 -80.7833 50 34.9 0.3 0.6

22-Jan-14 -3.3167 -80.7833 0 33.6 0.1 -0.3

22-Jan-14 -3.3167 -80.7833 10 33.8 0.4 0.3

22-Jan-14 -3.3167 -80.7833 20 34.4 0.3 0.5

22-Jan-14 -3.3167 -80.7833 30 34.7 0.3 0.7

22-Jan-14 -3.3167 -80.7833 40 34.8 0.3 0.7

22-Jan-14 -3.3167 -80.7833 50 34.9 0.4 0.9

22-Jan-14 -3.3333 -80.5833 0 33.6 0.1 -0.3

22-Jan-14 -3.3333 -80.5833 10 33.7 0.1 -0.3

22-Jan-14 -3.3333 -80.5833 20 34.3 0.4 0.1

22-Jan-14 -3.3333 -80.5833 30 34.6 0.2 0.6

22-Jan-14 -3.3333 -80.5833 40 34.7 0.3 0.6

22-Jan-14 -3.0833 -80.5833 0 33.5 0.2 -0.8

22-Jan-14 -3.0833 -80.5833 10 33.5 0.2 -0.7

22-Jan-14 -3.0833 -80.5833 20 34.0 0.3 0.0

22-Jan-14 -3.0833 -80.5833 30 34.7 0.3 1.0

22-Jan-14 -3.0833 -80.5833 40 34.8 0.1 0.4

25-Jan-14 -2.8333 -80.5833 0 33.5 0.0 -0.7

25-Jan-14 -2.8333 -80.5833 10 33.5 0.0 -0.4

25-Jan-14 -2.6000 -80.5833 0 33.6 0.0 0.1

25-Jan-14 -2.6000 -80.5833 10 33.8 0.0 -0.1

21-Jan-14 -3.1167 -80.3500 0 33.3 0.1 0.1

21-Jan-14 -3.1167 -80.3500 10 33.7 0.1 0.3

21-Jan-14 -3.1167 -80.3500 20 34.3 0.1 0.5

21-Jan-14 -3.1167 -80.3500 30 34.6 0.2 0.7

21-Jan-14 -3.1167 -80.3500 40 0.2 0.8

22-Jan-14 -3.3167 -80.3500 0 33.5 0.0 -0.4

22-Jan-14 -3.3167 -80.3500 10 33.7 0.1 0.0

22-Jan-14 -3.3167 -80.3500 20 34.1 0.2 0.5

18-May-14 -3.0611 -80.2197 0 29.6 -0.7 -3.8

18-May-14 -3.0611 -80.2197 5 30.1 -0.6 -3.0

18-May-14 -3.0611 -80.2197 10 31.6 -0.4 -2.3

18-May-14 -3.0611 -80.2197 15 32.2 -0.3 -1.3

18-May-14 -3.0833 -80.2197 0 28.6 -0.9 -4.8

18-May-14 -3.0833 -80.2197 5 30.5 -0.6 -2.8

18-May-14 -3.0833 -80.2197 10 31.8 -0.4 -1.9

18-May-14 -3.1067 -80.2197 0 29.5 -0.6 -4.0

18-May-14 -3.1067 -80.2197 5 30.8 -0.5 -2.8

18-May-14 -3.1067 -80.2197 10 32.1 -0.2 -1.5

18-May-14 -3.1067 -80.2197 15 32.7 -0.2 -1.3

18-May-14 -3.1300 -80.2197 0 30.1 -0.6 -3.5

18-May-14 -3.1300 -80.2197 5 31.2 -0.5 -2.5

18-May-14 -3.1300 -80.2197 10 32.1 -0.4 -1.7

18-May-14 -3.1517 -80.2197 0 29.6 -0.7 -4.1

18-May-14 -3.1517 -80.2197 5 31.2 -0.3 -3.5

18-May-14 -3.1517 -80.2197 10 32.7 -0.2 -1.8

18-May-14 -3.1750 -80.2197 0 28.6 -0.9 -6.3

18-May-14 -3.1750 -80.2197 5 30.3 -0.6 -3.7

18-May-14 -3.1750 -80.2197 10 32.8 -0.4 -1.8

18-May-14 -3.1978 -80.2197 0 29.6 -0.8 -4.4

18-May-14 -3.1978 -80.2197 5 31.1 -0.7 -2.8

18-May-14 -3.1978 -80.2197 10 33.2 -0.2 -0.7

18-May-14 -3.2200 -80.2197 0 29.7 -0.8 -4.2

18-May-14 -3.2200 -80.2197 5 30.0 -0.7 -4.0

18-May-14 -3.2200 -80.2197 10 33.3 -0.1 -0.4

18-May-14 -3.2433 -80.2197 0 30.2 -0.5 -4.0

18-May-14 -3.2433 -80.2197 5 30.6 -0.6 -3.4

18-May-14 -3.2433 -80.2197 10 32.5 -0.4 -1.1

18-May-14 -3.2647 -80.2197 0 30.2 -0.7 -3.7

18-May-14 -3.2647 -80.2197 5 31.4 -0.6 -2.8

18-May-14 -3.2647 -80.2197 10 32.9 -0.3 -1.0

18-May-14 -3.2647 -80.2197 15 33.5 -0.1 -0.3

18-May-14 -3.2889 -80.2197 0 31.3 -0.5 -2.5

18-May-14 -3.2889 -80.2197 5 31.3 -0.6 -2.7

18-May-14 -2.9950 -80.0750 0 29.9 -0.5 -3.5

18-May-14 -2.9950 -80.0750 5 30.2 -0.5 -3.4

18-May-14 -2.9950 -80.0750 10 30.8 -0.5 -2.6

18-May-14 -2.9950 -80.0750 15 31.7 -0.3 -2.0

18-May-14 -3.0083 -80.0583 0 29.7 -0.7 -3.8

18-May-14 -3.0083 -80.0583 5 29.6 -0.7 -4.0

18-May-14 -3.0083 -80.0583 10 29.8 -0.7 -3.7

18-May-14 -3.0083 -80.0583 15 31.2 -0.4 -2.2

18-May-14 -3.0083 -80.0583 20 31.4 -0.4 -2.3

18-May-14 -3.0233 -80.0417 0 28.2 -0.9 -5.3

18-May-14 -3.0233 -80.0417 5 28.8 -0.8 -4.8

18-May-14 -3.0233 -80.0417 10 29.5 -0.7 -4.2

18-May-14 -3.0400 -80.0267 0 28.3 -0.9 -5.4

18-May-14 -3.0400 -80.0267 5 28.9 -0.7 -4.9

18-May-14 -3.0544 -80.0083 0 25.1 -1.4 -8.3

18-May-14 -3.0544 -80.0083 5 25.9 -1.1 -9.2

18-May-14 -3.0700 -79.9933 0 26.0 -0.7 -10.0

18-May-14 -3.0700 -79.9933 5 27.0 -0.3 -9.7

18-May-14 -3.0867 -79.9767 0 26.3 -0.3 -10.9

18-May-14 -3.0867 -79.9767 5 26.5 -0.1 -11.1

18-May-14 -3.1017 -79.9600 0 26.2 -0.2 -11.0

18-May-14 -3.1017 -79.9600 5 26.3 -0.3 -10.8

19-May-14 -2.7433 -79.8967 0 18.9 -2.6 -15.9

19-May-14 -2.7433 -79.8967 5 19.9 -2.6 -15.3

19-May-14 -2.7433 -79.8967 10 22.2 -2.0 -11.3

19-May-14 -2.7433 -79.8967 15 22.9 -2.3 -13.2

19-May-14 -2.7433 -79.8811 0 20.2 -2.4 -14.3

19-May-14 -2.7433 -79.8811 5 21.8 -2.0 -12.6

19-May-14 -2.7433 -79.8811 10 22.5 -2.0 -11.5

19-May-14 -2.7433 -79.8644 0 18.0 -3.0 -17.2

19-May-14 -2.7433 -79.8644 5 18.8 -2.8 -16.0

19-May-14 -2.7433 -79.8644 10 20.4 -2.5 -14.1

19-May-14 -2.7433 -79.8478 0 18.7 -2.4 -14.5

19-May-14 -2.7433 -79.8478 5 19.8 -2.2 -13.4

19-May-14 -2.7433 -79.8333 0 19.2 -2.4 -14.3

19-May-14 -2.7433 -79.8333 10 20.4 -2.2 -12.7

19-May-14 -2.7433 -79.8133 0 13.7 -3.3 -20.8

19-May-14 -2.7433 -79.8133 5 15.5 -2.9 -18.3

19-May-14 -2.6617 -79.8333 0 12.6 -3.6 -22.3

19-May-14 -2.6617 -79.8333 5 14.8 -3.1 -19.7

19-May-14 -2.6617 -79.8333 10 16.4 -2.8 -17.8

19-May-14 -2.6617 -79.8133 0 12.7 -3.7 -22.4

19-May-14 -2.6617 -79.8133 5 13.1 -3.5 -21.8

19-May-14 -2.6617 -79.7972 0 8.1 -4.4 -28.2

19-May-14 -2.6617 -79.7972 5 12.4 -3.6 -22.7

17-May-14 -2.6967 -79.9600 0 18.9 -2.3 -14.3

17-May-14 -2.6967 -79.9600 5 20.0 -2.3 -12.9

17-May-14 -2.7000 -79.9631 0 20.0 -2.1 -13.0

17-May-14 -2.7000 -79.9631 5 19.9 -2.1 -13.2

17-May-14 -2.7050 -79.9600 0 19.3 -2.2 -13.9

20-May-14 -2.6383 -79.9333 0 15.5 -3.0 -19.2

20-May-14 -2.6383 -79.9333 5 17.5 -2.6 -16.6

20-May-14 -2.6383 -79.9100 0 15.6 -3.0 -18.8

20-May-14 -2.6383 -79.9100 5 17.4 -2.6 -16.8

20-May-14 -2.6383 -79.8933 0 14.1 -3.2 -20.8

20-May-14 -2.6383 -79.8933 5 15.7 -2.9 -19.3

19-May-14 -2.5856 -79.8217 0 8.9 -4.3 -27.4

19-May-14 -2.5856 -79.8217 5 10.1 -4.1 -25.9

19-May-14 -2.5856 -79.8111 0 8.9 -4.3 -27.3

19-May-14 -2.5856 -79.8111 5 10.1 -4.0 -26.0

20-May-14 -2.5222 -79.8800 0 6.1 -4.9 -31.3

20-May-14 -2.5222 -79.8800 5 6.4 -4.9 -30.8

20-May-14 -2.5222 -79.8739 0 6.2 -4.9 -31.0

20-May-14 -2.5222 -79.8739 5 6.9 -4.8 -30.0

20-May-14 -2.5222 -79.8667 0 5.3 -5.1 -32.1

20-May-14 -2.5222 -79.8667 5 7.4 -4.7 -29.5

19-May-14 -2.3578 -79.8483 0 0.2 -6.0 -38.0

19-May-14 -2.3578 -79.8483 5 0.2 -6.0 -38.8

20-May-14 -2.3578 -79.8378 0 0.2 -6.0 -38.6

20-May-14 -2.3578 -79.8378 5 0.2 -5.9 -38.8

20-May-14 -2.3578 -79.8300 0 0.2 -6.0 -38.6

20-May-14 -2.3578 -79.8300 5 0.2 -6.1 -38.7

19-May-14 -2.2467 -79.8756 0 0.2 -6.0 -38.0

19-May-14 -2.2467 -79.8756 5 0.2 -5.9 -38.3

20-May-14 -2.2467 -79.8600 0 0.1 -5.8 -36.5

20-May-14 -2.2467 -79.8600 5 0.1 -5.9 -36.8

20-May-14 -2.2467 -79.8381 0 0.1 -6.1 -38.9

20-May-14 -2.2467 -79.8317 0 0.1 -6.0 -39.0

16-May-14 -2.3567 -80.0150 0 19.7 -1.7 -9.9

16-May-14 -2.3567 -80.0150 5 19.6 -1.6 -10.0

16-May-14 -2.3567 -80.0150 10 19.6 -1.6 -9.8

16-May-14 -2.3617 -80.0083 0 19.6 -1.6 -9.9

16-May-14 -2.3617 -80.0083 5 19.7 -1.6 -9.6

16-May-14 -2.3617 -80.0083 10 19.8 -1.7 -9.5

16-May-14 -2.4522 -80.0583 0 20.4 -1.6 -9.2

16-May-14 -2.4522 -80.0583 5 20.4 -1.4 -9.4

16-May-14 -2.4567 -80.0483 0 20.2 -1.5 -9.3

16-May-14 -2.4567 -80.0483 5 20.1 -1.5 -9.2

16-May-14 -2.4600 -80.0400 0 19.0 -1.8 -10.3

16-May-14 -2.4600 -80.0400 5 19.3 -1.7 -10.3

16-May-14 -2.5250 -80.0978 0 20.3 -1.6 -9.4

16-May-14 -2.5250 -80.0978 5 20.4 -1.6 -9.5

16-May-14 -2.5350 -80.0817 0 20.6 -1.5 -9.3

16-May-14 -2.5350 -80.0817 5 20.5 -1.7 -9.2

16-May-14 -2.5483 -80.0633 0 14.0 -3.0 -18.1

16-May-14 -2.5483 -80.0633 5 17.7 -2.3 -14.2

17-May-14 -2.6500 -80.0889 0 20.8 -1.8 -10.8

17-May-14 -2.6500 -80.0889 5 20.8 -1.8 -10.6

17-May-14 -2.6628 -80.0867 0 19.8 -2.3 -12.7

17-May-14 -2.6628 -80.0867 5 19.7 -2.3 -12.6

17-May-14 -2.6744 -80.0847 0 19.0 -2.4 -13.7

16-May-14 -2.6817 -80.2450 0 23.8 -1.4 -8.1

16-May-14 -2.6817 -80.2450 5 23.8 -1.4 -8.0

16-May-14 -2.6817 -80.2450 10 23.8 -1.4 -7.9

16-May-14 -2.6989 -80.2294 0 21.3 -1.9 -10.4

16-May-14 -2.6989 -80.2294 5 21.7 -1.8 -10.0

16-May-14 -2.6989 -80.2294 10 23.2 -1.5 -8.7

16-May-14 -2.6989 -80.2294 15 23.5 -1.5 -8.3

16-May-14 -2.6989 -80.2294 20 23.5 -1.5 -9.0

17-May-14 -2.7117 -80.2167 0 17.4 -2.2 -13.5

17-May-14 -2.7117 -80.2167 5 20.9 -1.9 -11.5

17-May-14 -2.7117 -80.2167 10 21.5 -1.9 -11.3

17-May-14 -2.7500 -80.2833 0 26.5 -0.1 -9.6

17-May-14 -2.7500 -80.2833 5 26.7 -0.2 -9.3

17-May-14 -2.7700 -80.2722 0 27.6 -0.1 -8.4

17-May-14 -2.7700 -80.2722 5 27.5 -0.1 -8.5

17-May-14 -2.7700 -80.2722 10 27.7 0.0 -8.4

17-May-14 -2.7917 -80.2611 0 26.1 -0.3 -9.8

17-May-14 -2.7917 -80.2611 5 26.5 -0.2 -9.3

17-May-14 -2.7917 -80.2611 10 27.1 -0.6 -7.3

17-May-14 -2.8100 -80.2517 0 26.6 -1.3 -5.9

17-May-14 -2.8100 -80.2517 5 26.9 -1.2 -5.9

**Methods**

The water samples were collected using Van Dorn bottles lowered into the water with lines, typically from 9.5-m-long survey boats and at a few sites from bridges. Geographical coordinates of the boats were determined using C-Nav GPS systems. The Van Dorn bottles were triggered when they reached specified depths then brought back aboard the boats, where aliquots of water for salinity and stable isotope analyses were promptly put in 250 ml glass bottles and 20 ml glass vials, respectively, and sealed using conic plastic caps and electrical tape. These bottles were kept under refrigerated conditions prior to being analyzed.

The 250 ml bottles with Guayas estuary water samples were taken to the Department of Chemical Oceanography at the Instituto Oceanográfico de la Armada (INOCAR) in Guayaquil, Ecuador, where a HACH HQ14d meter with conductivity probe was used to determine their salinities. A water sample’s conductivity depends on the amount of dissolved salts in it and the temperature at which conductivity is measured. The response of the conductivity probe was calibrated by inserting it into solutions of known salinity and temperature, then the probe was inserted into aliquots of water from the Guayas estuary samples and their conductivity and temperature were measured. Based upon the established probe response, the salinity of each water sample was calculated from its measured conductivity and temperature. Standard procedures for instrument calibration and the direct measurement method were followed (for example, see document DOC316.53.01199 available from <https://www.hach.com/>).

To assess the consistency of salinity measurements, aliquots of a standard solution were periodically analyzed with the Guayas estuary samples. The estimated precision of an individual salinity analysis is ±0.1 on the PSS-78 practical salinity scale, as indicated by the standard deviation of the replicate analyses of standards. Replicate analyses of the salinities of several randomly selected Guayas estuary samples were also made. In each instance, the difference between each pair of measured values was smaller than the estimated precision of the individual measurements, so a single value for each sample is presented in Table 1.

The salinity values of discrete water samples determined in the laboratory were compared to *in situ* profiles of salinity obtained with a SeaBird CTD at the sites where the water samples were collected. The water sample values agreed well with the *in situ* profile values, indicating the Van Dorn bottles triggered at the intended water depths and the samples were not affected by handling prior to being analyzed.

The 20 ml vials with Guayas estuary water samples were taken to the Stable Isotope Geosciences Facility at Texas A&M University in College Station, Texas, USA. Their ^18^O/^16^O and ^2^H/^1^H ratios where measured using a Picarro L2120i cavity ring-down spectroscopy (CRDS) analyzer with a vaporization module. The near-infrared absorption spectrum of water vapor molecules depends upon their oxygen and hydrogen isotopic compositions. An auto-sampler delivered an aliquot of liquid water to CRDS system, where it was converted to vapor and its near-infrared absorption spectrum was determined at a constant temperature (e.g., method of Gupta et al., 2009).

Isotope measurements of Guayas estuary water samples were calibrated to the SIGF2013 and JGULF internal laboratory standards, which in turn, were calibrated using VSMOW, GISP, and SLAP standards. The oxygen and hydrogen isotopic compositions of the Guayas estuary water samples are expressed in δ notation relative to VSMOW (Table 1). Typically, two standards were analyzed with every seven Guayas estuary water samples. Analytical precisions of individual δ^18^O and δ^2^H analyses are ±0.1‰ and ±1.0‰, respectively, as estimated from the standard deviations of values obtained from replicate analyses of the internal laboratory standards.

**Ethics Statement**

Both authors contributed substantially to the production of this dataset, and it did not involve any human subjects or animal experiments.

**CRediT Author Statement**

Niall Slowey: conceptualization, methodology, resources, writing- original draft & revision. Edwin Pinto: conceptualization, methodology, resources, investigation, writing- review & editing.

**Acknowledgments**

We thank INOCAR and Texas A&M University for supporting the collection and analysis of the Guayas estuary water samples. In particular, we thank L. Burgos and other members of INOCAR’s Department of Chemical Oceanography as well as A. van Plantinga and K. Crabill of Texas A&M University for assistance and discussions.

**Declaration of Competing Interest**

The authors declare that they have no competing financial interests or personal relationships that have, or could be perceived to have, influenced the work reported in this article.

**References**

[1] E.B. Pinto & N.C. Slowey (2021) Stable isotope evidence for the origins of waters in the Guayas estuary and Gulf of Guayaquil. *Estuarine, Coastal and Shelf Science*, doi: https:// doi.org/10.1016/j.ecss.2020.107151.

[2] P. Gupta, D. Noone, J. Galewsky, C. Sweeney & B. Vaughn (2009) Demonstration of high-precision continuous measurements of water vapor isotopologues in laboratory and remote field deployments using wavelength-scanned cavity ring-down spectroscopy (WS-CRDS) technology. Rapid Communications in Mass Spectrometry, 23, 2534-2542.

[3] HACH Conductivity, Direct Measurement Method 8160. DOC316.53.01199_9ed.pdf From the Hach Water Analysis Handbook <https://www.hach.com/wah> (accessed 7 September 2020)
